# Supplementary material for: Gender differences in Dutch research funding over time: A statistical investigation of the innovation scheme 2012–2021
Source: PLoS One. 2024 Feb 16;19(2):e0297311. doi: 10.1371/journal.pone.0297311 (PMC10871518; doi:10.1371/journal.pone.0297311)
Supplement: S1 Appendix — (PDF) [file pone.0297311.s001.pdf]

S1 Appendix
Interpretation of the logistic regression coefficients

Although, as always in logistic models, the parameters in Table 4 cannot be directly interpreted, their sign and p-value can. To show how to use the numbers in Table 4, consider the following examples of a female and male applicant to the field ENW in 2020. For the female applicant, we have

$$\log\left(\frac{p_i}{1-p_i}\right) = -2.164 + 0.808 + 0.040 \times 10 = -1.038,$$

which corresponds to a success probability of 26.2%. For the male applicant we have

$$\log\left(\frac{p_i}{1-p_i}\right) = -2.164 + 0.417 + 0.808 + (0.040 - 0.094 - 0.391) \times 10 = -1.763,$$

corresponding to a success probability of 14.7%. For 2012, this domain had more balanced predicted success rates (20.5% for women, 20.9% for men; see Table 5).

From these (predicted) probabilities, the (predicted) odds ratio is computed as follows. First, probabilities are converted into odds via $odds = p/(1-p)$. Second, the odds ratio is computed by dividing the odds for male applicants by that for female applicants. E.g., for 2012 the predicted odds ratio is $(0.209/.791)/(0.205/.795) = 1.027$, as can also be seen in Table 7.
