# Supplementary material for: Gender differences in Dutch research funding over time: A statistical investigation of the innovation scheme 2012–2021
Source: PLoS One. 2024 Feb 16;19(2):e0297311. doi: 10.1371/journal.pone.0297311 (PMC10871518; doi:10.1371/journal.pone.0297311)
Supplement: S2 Appendix — (PDF) [file pone.0297311.s002.pdf]

S2 Appendix
Analysis code

```
venidata <- read.csv("venistats.csv", sep=";")
venidata$Field <- relevel(factor(venidata$Field), "SGW")
themodel1 <- glm(cbind(Granted, Applications - Granted) ~
    Gender + Field + Year, data = venidata,
    family = "binomial")
themodel2 <- glm(cbind(Granted, Applications - Granted) ~
    Gender*Year + Field , data = venidata,
    family = "binomial")
themodel3 <- glm(cbind(Granted, Applications - Granted) ~
    Gender*Year + Gender * Field , data = venidata,
    family = "binomial")
themodel4 <- glm(cbind(Granted, Applications - Granted) ~
    Gender*Year + Gender*Field + Year*Field, data = venidata,
    family = "binomial")

library("car")
Anova(themodel1, type = "III",test.statistic = "Wald")
Anova(themodel2, type = "III",test.statistic = "Wald")
Anova(themodel3, type = "III",test.statistic = "Wald")
Anova(themodel4, type = "III",test.statistic = "Wald")
AIC(themodel1,themodel2,themodel3,themodel4)
```
