# Supplementary material for: Gender differences in Dutch research funding over time: A statistical investigation of the innovation scheme 2012–2021
Source: PLoS One. 2024 Feb 16;19(2):e0297311. doi: 10.1371/journal.pone.0297311 (PMC10871518; doi:10.1371/journal.pone.0297311)
Supplement: Attachment — Submitted filename: responseletter.pdf [file pone.0297311.s003.pdf]

Response to the comments of the editor and reviewers

We thank the editor and reviewers for their careful reading of our manuscript and their helpful and constructive comments. Below, we will respond to the comments by the editor and the reviewers.
As we've typeset in the manuscript in LaTeX, there's no easy way to show where the paper has been revised (as the 'track changes' option in Word). We've indicated large changes/insertions in the text using blue text. Minor changes (such as corrections of spelling mistakes) have been made without indication in colour.

Responses to the comments of the editor

Comment:
In line/Besides reviewers' suggestions, consider the following recommendations:
1 Experiments, statistics, and other analyses are performed to a high technical standard and are described in sufficient detail.
Extend your argument on the potential relevance of institutional affiliation in the in the probability of success in your model (in "Model" or "Discussion" sections?)

Response:
We thank the editor for this suggestion. Unfortunately we do not have information on the institutional affiliation, so we are not able to take this into account. We have included this in the revised version of the manuscript. It is, however, important to note that institutional differences in the Netherlands are much smaller than quality differences between institutions in the UK and USA.

Comment:
Following good practices standards in statistical practice (https://www.nature.com/articles/s41562-021-01211-8) an additional section aimed to visualizing the data, besides the "Observed success probabilities" figures, could be added to excel the descriptive/explicative interpretation of results in logistic regression models, such as:
- Correlation Matrix
- ROC Curve
- Confusion Matrix

Response:
Thanks for pointing us towards this paper, of which the first author of the current manuscript is a co-author.

The suggested figures based on a correlation/confusion matrix or ROC curve won't work for this study. These tools are designed for 2×2 tables of the kind:

	Prediction: Yes	*Prediction: No*
Truth: Yes	TP (a)	FN (b)
Truth: No	FP (c)	TN (d)

where, ideally, the values on the diagonal (TP and TN), or a/(a+b) and d/(c+d), are as high as possible.

Our data, for a given field and year, is of the form:

	Gender: Male	*Gender: Female*
Funding: Yes	a	b
Funding: No	c	d

Here, a and d don't have the usual interpretation as True Positive and True Negative. If the values on the diagonal were both high, and the off-diagonal values low, it would be indicative of *large* gender differences, rather than of a desired situation.

Furthermore, the ROC curve for a 2×2 table would be a single point. The ROC curve becomes interesting once there is a (semi-)continuous threshold variable (such as dosage of a medicine, or number of questions of a test answered correctly), such that for each level of this threshold variable the sensitivity and specificity can be plotted. In this study, we do not have such a threshold variable.

In essence, our dataset is rather minimalistic: per gender, year and field it contains the total number of applications and number of granted applications. In the absence of more information, it is difficult to provide more visualisations, as suggested by the editor, without recycling the information which is already displayed in one of the figures. Based on Reviewer #1's suggestion to also study odds ratio's, we have added a Figure (Figure 3) on that topic. Given the relative simple format of the data, and the relative short length of the manuscript, we do not think it's necessary to move the figures to a dedicated section.

Comment:
Extend your conclusion section with:
- The paragraph in which it is stated that "more women than men leave academia before" is of great interest for the community of practice. Extend your conclusions on that.
- The limitations of the study.
- Further research agenda derivates from it.

Response:
We have incorporated changes in line with the requests from the editor. In the conclusion we have extended our conclusion on the fact that more women leave academia, and have included limitations and (connected to this) suggestions for future research.

Comment:
The article is presented in an intelligible fashion and is written in standard English. Extend the abstract content with the following criteria: “Background or Introduction – What is currently known? Start with a brief, 2 or 3 sentence, introduction to the research area. Objectives or Aims – What is the study and why did you do it? Clearly state the research question you’re trying to answer. Methods – What did you do? Explain what you did and how you did it. Include important information about your methods, but avoid the low-level specifics. Some disciplines have specific requirements for abstract methods. CONSORT for randomized trials. STROBE for observational studies PRISMA for systematic reviews and meta-analyses. Results – What did you find? Briefly give the key findings of your study. Include key numeric data (including confidence intervals or p values), where possible. Conclusions – What did you conclude? Tell the reader why your findings matter, and what this could mean for the ‘bigger picture’ of this area of research.”

Response:
We’ve rewritten the abstract in the indicated format.

Comment:
The article adheres to appropriate reporting guidelines and community standards for data availability.
Data and code for replication was submitted by the authors: https://osf.io/8bfaz/. Consider mention it in the manuscript body (“Data & materials” section?).

Response:
We’ve included the link to OSF in Footnote #2 (regarding data) and on page 10 (regarding the R code). For visibility, we’ve also included a ‘Data availability’ section at the end of the paper (page 12), but this can be omitted if this is preferred by the editor.

Responses to the comments of the reviewers

Comment:
1. Is the manuscript technically sound, and do the data support the conclusions?

Experiments must have been conducted rigorously, with appropriate controls, replication, and sample sizes. The conclusions must be drawn appropriately based on the data presented.

Response:
We thank the reviewer for the kind comment.

Comment:
2. Has the statistical analysis been performed appropriately and rigorously?
Reviewer #1: No
Reviewer #2: Yes

Response:
It seems that reviewer #1's "no" is fully due to an unfortunate sentence in which we interpret the p-value. We will address that comment under #5 below.

Comment:
3. Have the authors made all data underlying the findings in their manuscript fully available?
4. Is the manuscript presented in an intelligible fashion and written in standard English?

Reviewer #1: Yes [to both]
Reviewer #2: Yes [to both]

Response:
Thanks.

Comment (Reviewer #1):
5. Review Comments to the Author
Reviewer #1: As regards to form, the paper is fit for publication, and I have no comments whatsoever in that regard. However, I believe that two relatively important issues prevent it from being published in its actual form.

The first one, and probably the most important, relates to results interpretation. In the discussion section, the authors say: "In absence of any gender effects in the quality of applications and considerations of the assessment committee, the probability of finding at least the gender difference was smaller than 0.001, i.e. there is a very significant gender difference to the favour of women." While the first part of this

sentence is adequate, the last part is plain wrong. P-values in logistic regression are to be interpreted in the same way as in any other frequentist statistical test, that is, as the probability of obtaining results at least extreme under the hypothesis we aim to nullify. If the probability is lower than a specified threshold, we can safely reject that hypothesis, and that's it. P-values do not say anything about the significance or the plausibility of any other hypothesis, since the hypothesis we aim to nullify is the only one being tested. In order to say anything more, you need to measure effect size. We invite the authors to look at how this can be done as regards to logistic regression; as a suggestion, a thorough analysis of odds ratios should be appropriate.

Response:
There are various ways of frequentist null hypothesis significance testing. The reviewer advocates Fisher's approach in which the specific form of the alternative hypothesis is irrelevant. Our statement was in line with the approach by Neyman and Pearson. In the social sciences, the Neyman-Pearson approach, or the NHST amalgamation between both approaches, is more common than Fisher's approach. See, for instance, https://www.frontiersin.org/articles/10.3389/fpsyg.2015.00223/full for a thorough description of the differences and similarities between both approaches.

As the intention of our paper is not to have a fundamental debate about the philosophy of statistics, we have rephrased this debated sentence, such that it is acceptable to both those that follow Fisher's approach as those that follow the Neyman-Pearson approach, without adding statistical-philosophical details to the manuscript itself.

We have followed up the suggestion to present the odds ratios besides the predicted probabilities. We have added a paragraph on this on page 11, as well as Table 7 and Figure 3.

Comment (Reviewer #1):
The second point relates to a comment made at the beginning of the article, where the authors mention that "in certain situations, such as childcare responsibilities, terms [for the different funding programs] can be extended". Given the pervasive an systemic gender imbalance all over the world as regards to childcare, it is reasonable to hypothesize that this childcare extension could have been a major factor in improving the gender imbalance situation as regards to the Veni grants, especially since it is probably during that period the necessities of child caring places the greatest strain on female scholars' careers. We suppose that data regarding such extensions is not available as of now; if it were, I don't think this research would be complete or publishable without a thorough analysis of grant extensions. But the unavailability of such data doesn't make the case of childcare extensions for Veni grants less crucial to the general study of gender imbalances in research funding. In light of this, we believe it is imperative that the authors discuss this matter thoroughly, at the very least as an unfortunate and (perhaps) unavoidable limitation of their research.

Response:
NWO does offer extensions to the duration after graduation for which you're eligible for a grant, both maternal as paternal, but we indeed have no data on how many applicants made use of this. Furthermore, the regulations on these extensions are not static during the 2012-2022 period, and NWO only lists the current practice on their website.
We've now explicitly mentioned this in the discussion section ("We also lack the data on the number of applicants that received extensions due to childbirth and childcare and thus cannot measure the extent to which this affected the results.").
We also wish to re-iterate the next sentences in the discussion section: "The purpose of this paper is not to find the mechanisms behind observed gender effects, nor to state whether or not they are due to gender bias, but merely to answer the question whether the observed gender effects are statistically significant".
Finding the causal mechanisms behind the observed gender differences obviously is tremendously important, yet it's not the goal of this paper (nor is it possible to study this with the dataset at hand).

Comment (Reviewer #2):
Reviewer #2: The manuscript aims at analyzing gender differences in award rates for research grants of the Dutch Research Council (NWO; Veni, Vidi, Vici). In particular, the focus is on temporal developments: Do gender inequalities have changed over time? To do so, the authors use publicly available NWO data on submissions and awarded grants (by gender) for the years 2012 to 2021. The analyses are mainly based on logistic regressions with interaction terms to detect temporal changes of the gender effect. The key finding is that throughout the observation period the success probabilities of female applicants have increased compared to success rates of male applicants.
The authors convincingly show how their work adds to the literature. I think that the research question can be answered well with the data. The analyzes are well comprehensible and well documented so that replication is possible. Although the authors mainly intend to provide descriptions of developments, they also shortly discuss possible explanations for the observed results. The discussion of their results reveals that they are aware of the limitations of their study. I really appreciate the clear writing style.

Response:
We thank the reviewer for the friendly comments.

Comment (Reviewer #2):
A few more detailed comments:
Lines 66-68: I would not say that all models indicate lower success rates of males. For example, model 3 shows a positive gender (main) effect, meaning that at year=0 males have an advantage over females. By the way, I would recommend to code the variable "year" slightly different, so that the value zero refers to the first year in the data (i.e., 2012=0, … , 2021=9)

Response:
We have rephrased this as “For the Veni tier, all models find significant differences between the succes rates of male and female applicants. The models also show a trend over time, where male applicants gradualle have lower success rates and female applicants gradually have higher success rates.”
The new Figure 3 also demonstrates the effects we find nicely: while Model 1 (which does not assume temporal effects) provides an odds ratio < 1 (indicating better success rates for females), the other models show a decline in OR over time, where for various fields the date where the curve crosses the OR = 1 line (e.g. swapping from male advantage to female advantage) varies.
We followed the suggestion to recode “year”, such that counting starts with 0 instead of 1. As a consequence, some values in the manuscript (especially in Tables 2 and 4) have changed, but all interpretations, effect sizes and p-values remain unchanged.

In revising Table 2, we found out that in the original manuscript the values for ‘Field’ and ‘Year’ were accidentally swapped for Models 2, 3 and 4. We have corrected this now. The text was written based on the correct tables, so no textual changes were necessary due to this mistake.

Comment (Reviewer #2):
Line 77: As far as I know Lutter & Schröder do find evidence for gender inequalities controlling for publication output (in favor of female researchers).

Response:
The reviewer is completely right, it was an error because of the way the statement was framed. What we meant to say is that Lutter and Schroder do not find evidence for a male premium in hiring probability, but instead the opposite. This has been changed in the revised version of the manuscript.
